# Surface Topography Analysis of BK7 with Different Roughness Nozzles Using an Abrasive Water Jet

**DOI:** 10.3390/ma17184494

**Published:** 2024-09-13

**Authors:** Haihong Pan, Xuhong Chen, Lin Chen, Hui You, Xubin Liang

**Affiliations:** Precision Polishing and Measuring Laboratory, Guangxi University, Nanning 530000, China; hustphh@163.com (H.P.); 18172423561@163.com (X.C.); hyou@gxu.edu.cn (H.Y.); gxnnlxbwlh@163.com (X.L.)

**Keywords:** nozzle roughness, AWJ, surface topography (RMS,PV), BK7 roughness, abrasive wear, ANOVA

## Abstract

This study investigated the effect of abrasive water jet (AWJ) kinematic parameters, such as jet traverse speed and water pressure, abrasive mass flow rate, and standoff distance on the surface of BK7. Nozzle A reinforced with a 100 nm particle-sized coating of titanium alloy has more wear resistance compared to Nozzle B coated with nothing. Through analysis of variance and measurement of BK7 surface quality, it is concluded that the grooving and plowing caused by abrasive particles and irregularities in the abrasive water jet machined surface with respect to traverse speed (3, 7.2, 7.8, and 9 mm/min), abrasive flow rate (7 L/min and 10 L/min, 80 mesh) and water pressure (2 and 3 MPa) were investigated using surface topography measurements. The surface roughness (15.734 nm) of BK7 results show that a nozzle coated with titanium alloy has more hardness, which protects BK7 undamaged and super-smooth. The values of selected surface roughness profile parameters—average roughness (Ra) and maximum height of PV (maximum depth of peak and valleys)—reveal a comparatively smooth BK7 surface in composites reinforced with 2% titanium alloy in the nozzle weight at a traverse speed of 7.8 mm/min. Moreover, abrasive water jet machining at high water pressure (3 MPa) produced better surface quality due to material removal and effective cleaning of lens fragmentation and abrasive particles from the polishing zone compared to a lower water pressure (2 MPa), low traverse speed (5 mm/min), and low abrasive mass flow rate (200 g/min).

## 1. Introduction

In recent years, water jet theory and techniques have been extensively studied and explored by a wide range of scholars. To solve certain challenges in manufacturing operations, which include machining high-strength and damaged and cracked work pieces to produce ultra-smooth surfaces with the required accuracy with respect to miniaturization, waste reduction, and energy conservation, unconventional technologies are used. The essence of these technologies is the use of energy (Mechatronics, chemical or Mechatronic hydraulic mixing) rather than a solid cutting tool and grinding as used in conventional machining. However, the unique characteristics of the material, including its micro-structure and non-conventional thermal processes are no longer appropriate for some materials.

Jet polishing technology is an efficient and non-contact optical component processing method widely used for surface polishing of optical lenses, crystals, and other materials. However, nozzle wear is a common problem in jet polishing processes, especially for optical glass materials such as BK7. BK7 belongs to amorphous optical materials and its structure belongs to polar polymer condensed matter. Its interior presents a short-range ordered and long-range disordered amorphous structure. Li, Z.B. et al. optimized the nozzle section geometry, the cutting capacity, and the life cycle of the nozzle [1]. Oka, Y. et al. [2,3,4,5,6] studied the impact damage of abrasive particles on materials, systematically analyzed the effects of abrasive particle properties, impact parameters, and physical and chemical characteristics on erosion, and established relevant prediction equations for subsequent studies of nozzle runner erosion. On this basis, the nozzle runner erosion was studied extensively. Bozzini, B. et al. [7] listed some main factors affecting the erosion rate, including flow velocity, sand content, sand size, impact angle, liquid pH value, temperature, oxygen content, etc. Stack, M.M. et al. [8] studied material erosion and discussed the relative advantages and limitations of CFD modeling with erosion mapping to model the erosion behavior of pure metals. Long, X. et al. [9] analyzed a decrease in particle shape factors, which include particle density and particle diameter. They numerically simulated the internal flow and particle motion of an abrasive jet nozzle and calculated the motion trajectory of abrasive particles by using DPM. Kartal, F. [10] found four main factors of rotational speed, the nozzle forward speed of the nozzle, the abrasive flow rate speed, and the nozzle approach distance to achieve a smoother macro surface while using erosion resistance nozzles. From an angle of 30 degrees, the nozzle turns into a fluid jet tool and removes material independently [11]. Anand, U et al. [12] designed the lubricating nozzle, which has a harder surface to prolong the life cycle of the nozzle, but the process of fabricating the nozzle is complicated and the stability of the performance needs to be optimized. Schnecken, B. et al. [13], who predicted that more and more sensors should be prepared in the process (e.g., density sensor, flow sensor, pH sensor) in AWJ, built a static test set-up and tested slurry erosion in glass polishing and the influence of slurry erosion by conventional polishing nozzles. Hashish, M. [14] designed an empirical model for nozzle weight loss rate to analyze the wear mechanism of nozzles and concluded that the nozzle length, inlet angle, orifice diameter, abrasive flow rate, and water pressure on the nozzle had big effects on the nozzle life cycle. Shao, C. et al. [15] concluded that the particle impact position moves forward as the particle incident position moves away from the inlet center and the erosion rate was most sensitive to pump pressure. Chen, X. et al. [16] set up the pre-mixed AWJ with a nozzle inlet angle of more than 30 degrees and concluded that nozzle structure can reduce the erosion ratio by more than 88.14%. Flow profile, erosion rate, and erosion pattern are established to analyze the evolution of material surface induced by particle erosion [17]. A modified model for erosion was developed that accounts for the effect of particle size to simulate the wall impact velocity caused by fluid turbulence. It is demonstrated that, when compared to the previous simplified erosion models, the new model can estimate erosion rate more accurately, especially for small particles in gas–solid flows [18]. Zuo, W.Q. et al. [19] designed the dual gradient nozzle structure and studied abrasive diameter, abrasive particle density, and abrasive particle jet flow rate influencing the focus tube. Du, M.M.et al. [20] analyzed the nozzle wear pattern carefully and found most serious wear happened at the junction of the mixing chamber. The primary aim of this paper is to point out and raise awareness of the problem of slurry erosion and the influence of slurry erosion by conventional polishing nozzles. 

## 2. Experimental Facilities and Technical Parameter

### 2.1. Nozzle Property

**Figure 1:**
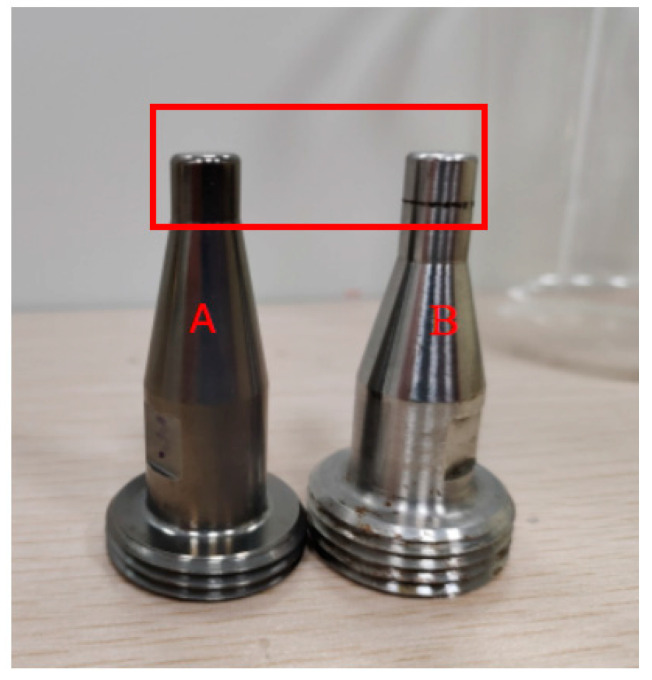
Physical image of nozzles.

Nozzle A is composed of 98% 304 stainless steel and 2% titanium, supplied by Liuzhou Yuanchuang Electronic Spray Technology Co., Ltd. (Liuzhou, China). Titanium alloy nanoparticles, approximately 100 nm in size, are used to reinforce Nozzle A. This hybrid casting process combines conventional casting and spraying coating techniques. It involves adding titanium alloy particles to the surface of nozzles using spraying methods. The machining process for Nozzle A includes the following five steps: machining the blank, electro-chemical deburring, inner hole polishing, inner hole coating, and passivation treatment on the outer surface of the nozzles. In contrast, the machining process for Nozzle B includes the following four steps: machining the blank, electro-chemical deburring, inner hole polishing, and inner hole coating. As mentioned above, Nozzle A has one additional processing step compared to Nozzle B. The physical images of Nozzle A and Nozzle B are shown in Figure 1. The titanium alloy percentage and surface properties of the different nozzles (Nozzle A and Nozzle B) are listed in Table 1.

**Figure 2:**
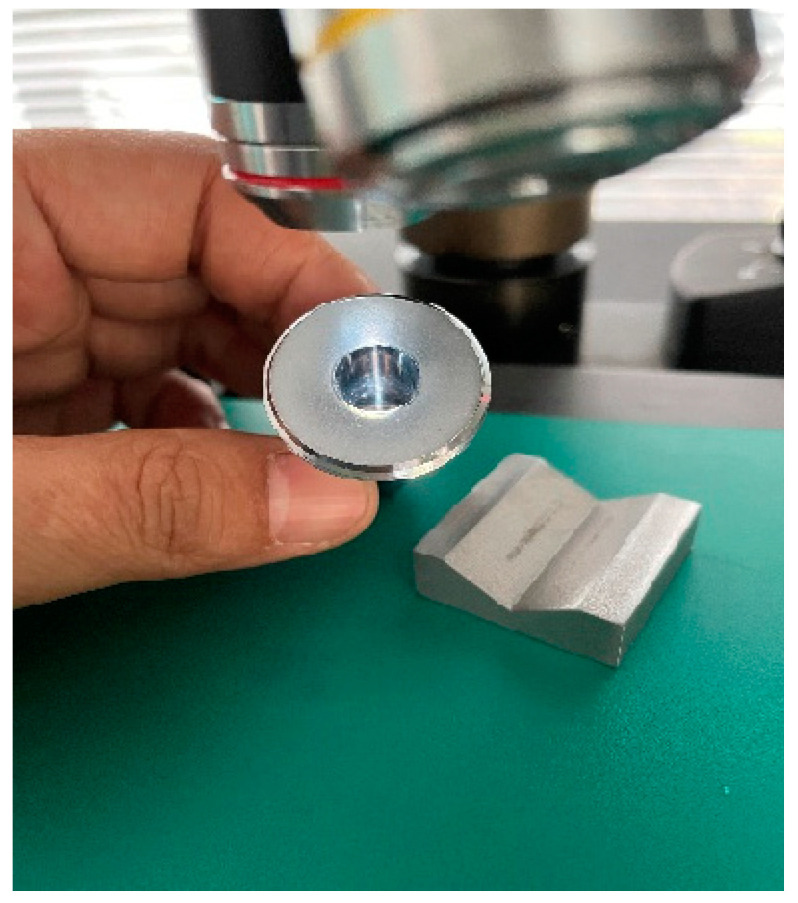
Surface roughness detection of nozzles (A and B).

The AWJ machining process is performed on a SUN 5-axis CNC machine. The machining process includes the following four steps: machining blank, electro-chemical treatment of burrs, inner hole polishing, and inner hole coating. The roughness of the nozzles are tested by a portable roughness instrument (Mitutoyo SJ210, Mitutoyo, Kawasaki, Japan), as shown in Figure 2. The coaxiality level of all surfaces is also obtained by a detector according to the GB/T 1184-1996 standard.

### 2.2. Performance and Experimental Dimensions of BK7

**Figure 3:**
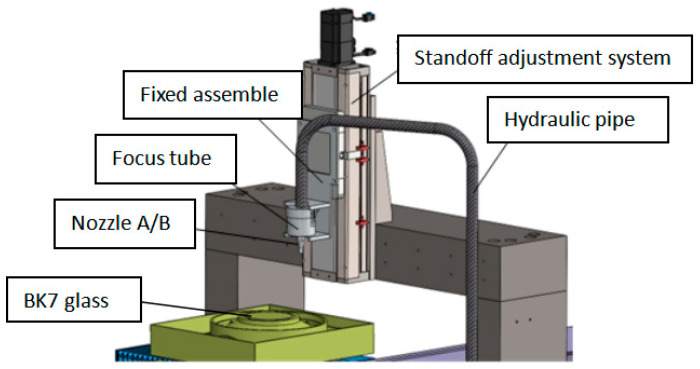
3D schematic diagram of AWJ.

**Figure 4:**
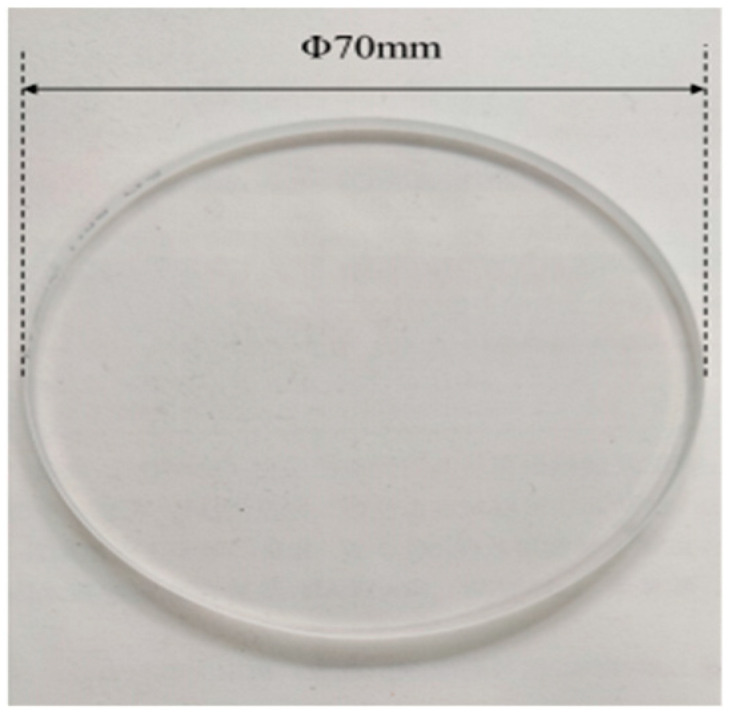
Polishing optical glass (BK7).

In continuous polishing, the simplified removal quantity model is expressed as a function relationship between the depth and width of the removal function and the velocity of the workpiece relative to the nozzle during the polishing process. To obtain the model, the experimental data fitting method is also used. The experimental platform, samples, and other experimental conditions are the same as point-by-point polishing, while the polishing speed increases rapidly from 0.01 mm/s to 0.24 mm/s. The three-dimensional simplified model of the polishing system equipment is shown in Figure 3. The experiment is conducted on the surface of BK7 optical glass with a thickness of 5 mm and diameter of φ70 mm, which is shown in Figure 4.

**Figure 5:**
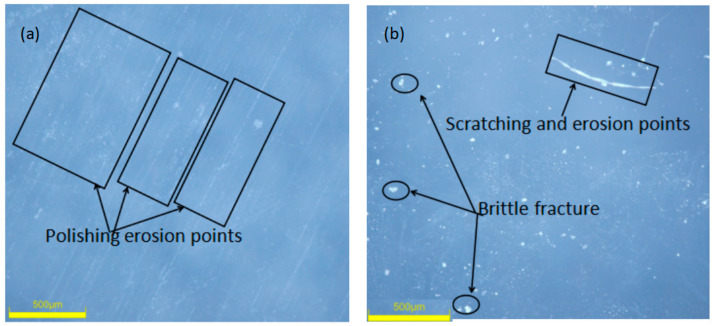
Optical images of polished BK7 surfaces using Nozzle A and Nozzle B, respectively. (**a**) The surface condition of BK7 glass after 500 h of testing with Nozzle A. (**b**) The surface condition of BK7 glass after 500 h of testing with Nozzle B.

From the microscopic image (Figure 5), it can be observed that the crack types of BK7 are mostly herringbone cracks and Y-shaped cracks. Arai, S. et al. [21] mentioned the “core” and “boundary” porosity in their study and confirmed that polishing can cause sub-surface damage to the surface layer of optical components.

## 3. Experimental Results and Discussion

### 3.1. Preliminary Experiments of AWJ

**Figure 6:**
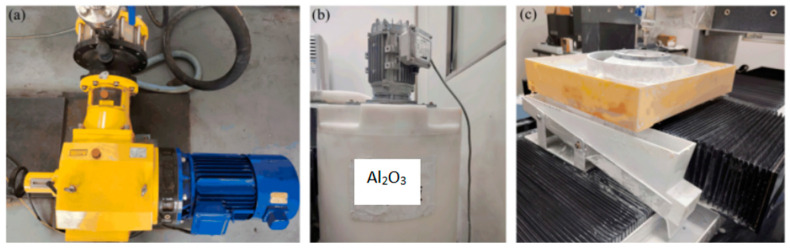
Experiment Equipment of AWJ. (**a**) Diaphragm pump. (**b**) Abrasive mixing system. (**c**) Polishing system.

The AWJ polishing process is carried out on a self-developed air flotation machine, which is shown in Figure 6c. The polishing lathe consists of three axes (X, Y, Z), which are equipped with one linear motor, respectively. The maximum speed of the linear motor can reach 1000 mm/s and the maximum acceleration can reach 10 kg. The continuous thrust on the X-axis is 875 N, with a peak thrust of 2469 N, and the continuous thrust on the Y-axis is 362 N, with a peak thrust of 1023 N. The technological conditions of experiments are shown in Table 2. In abrasive water jet polishing, there is no mechanical processing force between the nozzle and the workpiece, so the force of the motor is entirely used to overcome the frictional force generated by the workbench. So, the selection of the feed system motor can meet the design requirements.

By determining the value for achieving BK7 polishing, the process parameter combination parameters are selected, which are 3 MPa and a polishing medium flow rate of 100 mL/min. According to the polishing requirements, the supply pump is Shanghai Suneng SN-JYDR, which is shown in Figure 6a with a maximum pressure of 5 MPa and a maximum flow rate of 32 L/min. In the system, a stirrer was also added to the storage bucket, as shown in Figure 6b, which was used to prevent abrasives from settling in water and to make the polishing solution mix more evenly through continuous stirring. A polishing solution recovery system was also designed, as shown in Figure 6c, which consists of a polishing solution collection tank and a reflux pipe. The purpose of the design is to recover the polishing solution after polishing. After processing, we used a non-contact laser interferometer (ZYGO VeriFire, Middlefield, CT, USA) to check the 3D image of the BK7 polishing surface. We also used a super depth of field microscope to check the degree of scratches on the BK7 glass lens. 

### 3.2. Analysis of Variance

Azmir, M.A. et al. [22] studied the process parameters in polishing BK7 and found hydraulic pressure, abrasive mass flow rate, standoff distance, and traverse rate improved both criteria of machining performance. In order to comprehensively analyze the technical factors of processing BK7, this article uses statistical analysis of variance to analyze the important technical factors that affect the control of BK7 surface integrity. The appropriate combination of stable polishing force and polishing distance can improve surface roughness in ultra-precision polishing, as these conditions can produce smoother optical surfaces. When determining the main influencing factors for polishing BK7 optical glass, we use a statistical array of L16 (2^15^). To complete the statistical array, 16 machining tests, representing all combinations of interest, were evaluated with Nozzle B. Sixteen additional tests were conducted with Nozzle A (304 stainless steel/2% titanium). To achieve thoroughness, each test was repeated three times, using different BK7. Therefore, the subsequent analysis was based on our observations of 96 machined surfaces. In practice, this approach will highlight possible effects due to external variables. It is designed for polishing evaluation. For a series of statistical polishing experiments, 15 defining factors are as follows: A—feed rate (7.8 and 14.4 mm/min), B—abrasive flow rate (10 m/s and 5 m/s), C—polishing force, D—polishing particle size 1 μm and 500 nm, F—working polishing depth (50 nm and 25 nm), and G—polishing distance. A×B, A×C, B×C, A×B×C, A×D, A×C×D, A×G, C×F, A×C×G. Table 3 shows the selected factors and their specified levels in the analysis of variance. This method is adopted to consider the impact of environmental control on the polishing process. Each polishing experiment used aluminum oxide. In order to complete the statistical array, 16 processing experiments were conducted, representing all combinations of interest. Each experiment was repeated 3 times, using different BK7 specimens, followed by our observation of 96 machined surfaces. In practice, the ZYGO laser interferometer is used to measure (a) surface roughness and (b) surface smoothness of BK7 samples. Table 4 summarizes the statistical evaluation results of the polishing experiment. Emphasis was placed on the benefits of effective environmental control in ultra-precision polishing processes, in which case it is used to achieve continuous elimination of material removal points. 

### 3.3. Mechanical Properties Analysis of Nozzles

This paper mainly studies the surface morphology evolution of two nozzles with different surface materials after 500 hours of ultra-precision polishing. The roughness changes in the two nozzles after flowing by abrasive particles are shown in Table 5. By conducting 96 experiments on nozzles A and B, the cavity roughness of the original nozzles compared to the polished ones is averaged. The roughness of Nozzle A and Nozzle B are tested by a portable roughness instrument and flash tester. It is shown in Table 5 that after being impacted by abrasive particles, both Nozzle A and Nozzle B experience varying degrees of wear. It has been determined that coating with a lower titanium alloy shows 304 stainless steel delayed deformation and performed better [23].

According to Table 5, the range of roughness variation, which belongs to Aperture Ø 1’s (mm) outlet of Nozzle A, is from 1.057 mm to 1.145 mm, while Aperture Ø 1’s (mm) outlet of Nozzle B is from 1.092 mm to 1.692 mm. With the increase in titanium, the surface roughness change in Nozzle A is one-third less than that of Nozzle B without titanium alloy infiltration. This may be due to the fact that during the abrasive water jet processing, the surface of Nozzle A is coated with titanium alloy, and the titanium alloy particles are more than 10 times smaller than the abrasive particles, and the number of particles protects the surface from the impact of large abrasive particles [24]. Because the nozzle cavity maintains a good cone structure, when the abrasive flow through the nozzle always maintains a stable and continuous flow, it is not difficult to understand that a stable and continuous flow can ensure the improvement of the surface accuracy of the workpiece BK7. Hence, the increase in particle content in Nozzle A can protect its machined surface from damage. According to the surface roughness of nozzles A and B in Table 3 and Table 4, when the abrasive particles are mixed with water into the nozzle cavity, the surface damage caused by abrasive particles on the nozzle cavity can be reduced due to the titanium alloy plating on the nozzle cavity.

**Figure 7:**
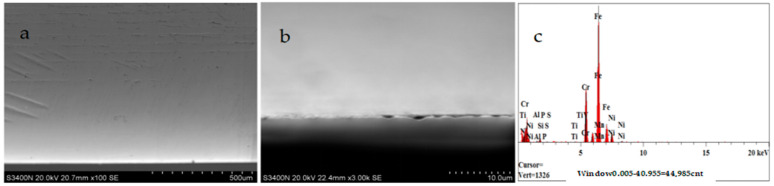
SEM images of Nozzle A coated titanium alloy composite after 500 h tests: (**a**) the wear area at the Nozzle A exit position, magnification 100×; (**b**) the wear area at the Nozzle A exit position, magnification 3000×; and (**c**) the main metal contents of the Nozzle A outlet wear area.

**Figure 8:**
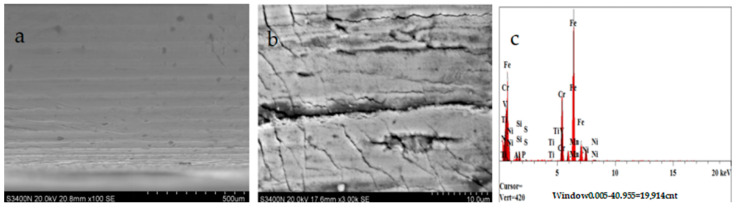
SEM images of Nozzle B without coating after 500 h tests: (**a**) the wear area at the Nozzle B exit position magnification 100×; (**b**) the wear area at the Nozzle B exit position magnification, magnification 3000×; and (**c**) the main metal contents of the Nozzle B outlet wear area.

The surface topography of the nozzle in this experiment was detected by Hitachi S-3400N electron microscopy. According to the test results, the wear problem of a nozzle without titanium alloy coating on the cavity surface is obviously higher than that of nozzles with titanium alloy coating on the surface, especially the wear problem of the nozzle outlet body as shown in Figure 7 and Figure 8. The Vickers hardness (HV) of 304 stainless steel is within 200 titanium alloy, while the main metal material is TiN, whose Vickers hardness can reach at least 800 or so. Hence, it seems reasonable that the wear resistance of Nozzle A is higher than that of Nozzle B in Figure 7a and Figure 8a. In the local magnification of nozzle wear in Figure 7b and Figure 8b, the surface topography of Nozzle A can also be seen due to the surface topography of Nozzle B. This result can also be proved by Table 5. The schematic diagram of metal contents in the wear zone of the nozzle (A, B) outlet is shown in Figure 7b and Figure 8b, respectively. Table 6 lists the percentage of metal content in the wear zone at the exit of nozzles A and B.

### 3.4. Surface Roughness and PV of BK7

The roughness parameters Ra and PV (maximum depth of valley and peak) were used to analyze the surface characteristics of BK7. The BK7 optical glass with similar initial surface morphology was polished using Nozzle A and Nozzle B, respectively. The PV values and RMS values before and after 48 group experiments of each nozzle (Nozzle A or Nozzle B) for polishing BK7 were plotted in Table 3, and the specific data were shown in Table 4. As shown in Table 7, after averaging the PV value and RMS value of the optical glass surface before and after the polishing tests, it can be seen that Nozzle A decreased from 318.765 nm before being polished to 160.135 nm after being polished, a decrease of 49.5%, and the RMS value decreased from 70.586 nm to 15.734 nm, a decrease of 80%. After polishing Nozzle B, the PV value of the glass surface decreased from 315.556 nm to 190.568 nm, with a decrease of 39.6%, and the RMS value decreased from 76.556 nm to 58.544 nm, with a decrease of 23.6%. According to the above data, the PV value and RMS on the optical glass surface are worth improving, and Nozzle A is better than Nozzle B.

**Figure 9:**
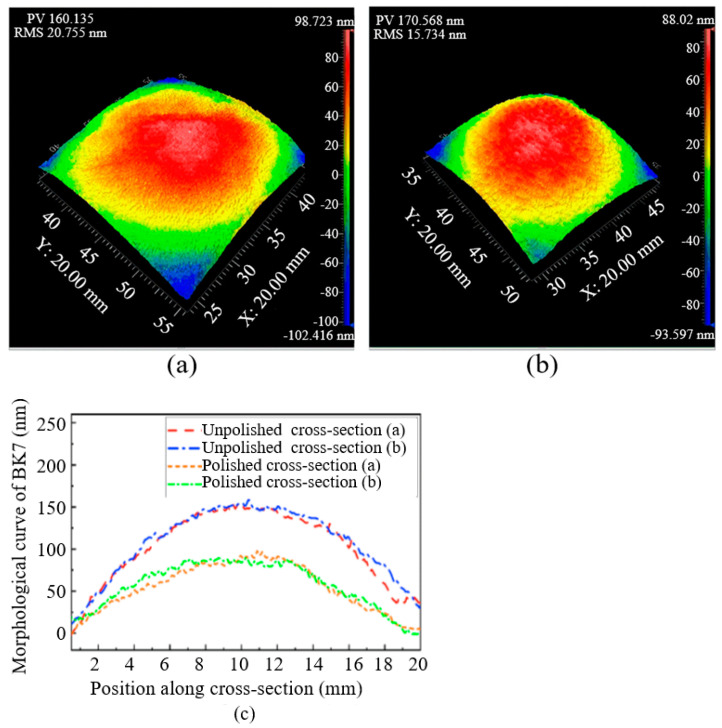
Optical quality of BK7 after Nozzle A polished: (**a**) 7.8 mm/min; 10 L/min; 500 nm grit size; hydraulic pressure 2 MPa; (**b**) optical quality of BK7 after polished: 7.4 mm/min; 20 L/min; 500 nm grit size; hydraulic pressure 3 MPa; (**c**) a graph in two orthogonal sections of BK7.

**Figure 10:**
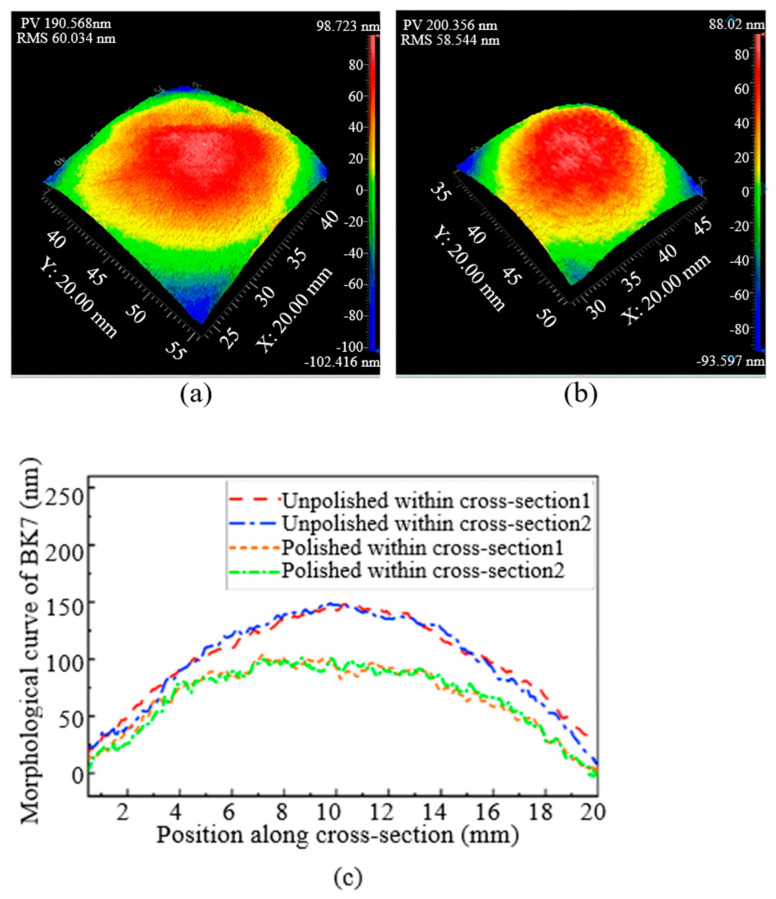
Optical quality of BK7 after Nozzle B polished: (**a**) 7.8 mm/min; 10 L/min; 500 nm grit size; hydraulic pressure 2 MPa; (**b**) optical quality of BK7 after polished: 7.4 mm/min; 20 L/min; 500 nm grit size; hydraulic pressure 3 MPa; (**c**) a graph in two orthogonal sections of BK7.

The PV and RMS values in Table 7 are the results of the optimal factor combination in the orthogonal experiment. Since each experimental combination was repeated three times, a total of three experimental results are listed. Through variance experiments, we obtained the optimal roughness and surface finish values for two types of nozzles with four optimal factor combinations. The topography of BK7 polished by Nozzle A and Nozzle B are shown in Figure 9 and Figure 10 in order to analyze the changes in machining parameters and nozzle roughness on the surface morphology of BK7 optical glass. Figure 9 and Figure 10 show the measurement results of the polished BK7 optical glass surface using a laser interferometer. It can be seen from the BK7 optical glass morphology that the PV value and RMS value of the optical glass surface can be significantly improved after polishing with nozzles A and B, thus improving the surface accuracy. However, it is also not difficult to find in Figure 9 and Figure 10 that the topography of BK7 polished by Nozzle A is better compared to Nozzle B.

## 4. Conclusions

Based on the experimental results, analysis of variance (ANOVA), and the effect of machining parameters on surface roughness and PV values, a practical analysis of machining with AWJ can be obtained, and the following conclusions can be drawn:

(1) Continuous and stable injection pressure (MPa) and abrasive material size (alumina) are considered to be the most significant control factors affecting the surface roughness of BK7.
(2) Reducing the injection distance and reducing the feed speed of the machine can improve the surface roughness and surface finish of the workpiece.
(3) The surface titanizing nozzle has better corrosion resistance and anti-beating ability and can maintain stable liquid pressure. It can be seen from this paper that the wear resistance of the stainless steel nozzle with a titanized surface is three times higher than that of the nozzle without a titanized surface. The working life cycle of the titanium alloy nozzle is two times higher than that of the nozzle without titanium.
(4) ZYGO test results showed that after 500 h of the nozzle, the surface roughness and surface finish of the processed BK7 work-piece decreased, which was caused by the unstable jet penetration process, uneven abrasive distribution in the jet and fluid divergence resistance at the exit of Nozzle B caused by wear.

Through variance analysis, the design effect of nozzle optimization has been identified as an important factor that can help improve surface smoothness. Therefore, titanium alloy infiltration on the nozzle surface is crucial for achieving the surface integrity of BK7.

In order to reduce the surface accuracy of BK7 caused by nozzle wear in jet polishing, the future development direction is mainly focused on nozzle material optimization, design improvement, polishing process optimization, real-time monitoring and control, green environmental protection, and intelligent polishing systems. Through these measures, it is expected to improve the stability and reliability of jet polishing technology, providing a more efficient and environmentally friendly solution in the field of optical component processing.

## Figures and Tables

**Table 1:** Titanium alloy percent and surface property of different nozzles.

Nozzle Composite (wt.%)	Titanium Alloy (wt.%)	Reinforcement (Thickness)	Reinforced Particles Size (nm)	Surface Roughness (μm)	Surface Hardness (HV)
Nozzle A: (304 stainless steel/2% titanium alloy)	2%	0.1 mm	100	0.041	700 HV
Nozzle B: (304 stainless steel)	N/A	N/A	N/A	0.041	200 HV

**Table 2:** Technological conditions of experiments.

Parameters	Symbols	Unit	Value
Test hours	h	hour	500
Water pressure	p	MPa	Variables 2, 3
Traverse speed	vt	mm/min	Variables 0.1, 7.2, 7.8, 14.4
BK7 thickness	h	mm	5
Diameter of BK7	d	mm	φ70
Mass flow rate of mixed liquid	v	L/min	Variables 10, 20
Abrasive size	-	mesh (mm)	80 (0.177)
Diameter of nozzle	do	mm	1
Diameter of focusing tube	df	mm	10
Stand-off distance	z	mm	10
Position of cutting head	j	°	90
Abrasive particle diameter	-	1000 nm,500 nm	Al_3_O_2_

**Table 3:** Statistical array L16 (215) designed for polishing experiments.

No.	Feed Rate (mm/min)	Abrasive Flow Rate m/s	Hydraulic Pressure	A×B	A×C	B×C	A×B×C	Abrasive Grit Size (μm)	A×D	Polishing Depth of Sample (nm)	A×B×D	Polishing Standoff Distance	C×G	C×F	A×C×G
1	7.8	10	2	+	+	+	+	0.5	+	50	+	2.5	+	+	+
2	7.8	10	2	+	+	+	+	1	−	25	−	2.5	−	−	−
3	7.8	10	3	+	−	−	−	0.5	+	50	+	5	−	−	−
4	7.8	10	3	+	−	−	−	1	−	25	−	5	+	+	+
5	7.8	5	2	−	+	−	−	0.5	+	50	−	2.5	+	−	−
6	7.8	5	2	−	+	−	−	1	−	25	+	2.5	−	+	+
7	7.8	5	3	−	−	+	+	0.5	+	50	−	5	−	+	+
8	7.8	5	3	−	−	+	+	1	−	25	+	5	+	−	−
9	14.4	10	2	−	−	+	−	0.5	−	50	−	2.5	−	+	−
10	14.4	10	2	−	−	+	−	1	+	25	+	2.5	+	−	+
11	14.4	10	3	−	+	−	+	0.5	−	50	−	5	+	−	+
12	14.4	10	3	−	+	−	+	1	+	25	+	5	−	+	−
13	14.4	5	2	+	−	−	+	0.5	−	50	+	2.5	−	−	+
14	14.4	5	2	+	−	−	+	1	+	25	−	2.5	+	+	−
15	14.4	5	3	+	+	+	−	0.5	−	50	+	5	+	+	−
16	14.4	5	3	+	+	+	−	1	+	25	−	5	−	−	+
Ref.	A	B	C	A×B	A×C	B×C	A×B×C	D	A×D	F	A×C×D	G	A×G	C×F	A×C×G
	1	2	3	4	5	6	7	8	9	10	11	12	13	14	15

**Table 4:** ANOVA statistical evaluation of polishing parameters for both Nozzle A and Nozzle B in summary, showing the analysis of variance for surface roughness of BK7, PV of BK7, and surface roughness of nozzles.

Factor	BK7 and Nozzle A (304 Stainless Steel/2% Titanium)			BK7 and Nozzle B (304 Stainless Steel)		
	Surface Roughness of BK7	PV of BK7	Surface Roughness (Nozzle A)	Surface Roughness of BK7	PV of BK7	Surface Roughness (Nozzle B)
Feed rate A	**13.35**			**23.35**	**22.12**	
Abrasive flow rate B	2.61	1.32	**40.35**	5.61	5.32	**25.35**
Hydraulic pressure C	**40.38**	**28.58**	**28.53**	**20.65**	10.28	**50.58**
Abrasive grit size D	**23.35**	15.23	11.12	**18.35**	8.23	10.37
Polishing depth F	4.13				8.85	
Polishing standoff distance G	10.56	2.11		5.56	7.61	
A×B		12.35			**13.35**	
A×C						
A×D						
A×G						
B×C			**20**			**14**
C×F					5.15	
A×B×C		**23.63**		12.45		
A×C×D	5.62	**16.88**		8.67	**12.34**	
A×C×G				5.36	7.25	
Error	0.92	0.15	0.10	1.82	0.3	0.15
Total (%)	100	100	100	100	100	100

Level of factors: bold/level 1.

**Table 5:** Geometric tolerances and roughness of different nozzles.

Inspected Items	Inspection Tool	Cavity Roughness of Nozzle A (μm)		Cavity Roughness of Nozzle B (μm)	
		Before Polished	After Polished	Before Polished	After Polished
Large end surface roughness Ra (μm)	Portable roughness instrument	0.117	0.205	0.156	0.455
Small end surface roughness Ra (μm)	Portable roughness instrument	0.072	0.188	0.084	0.658
Ø 9.5 hole roughness Ra (μm)	Portable roughness instrument	0.038	0.165	0.043	0.246
Aperture Ø 1 (mm) outlet of nozzle	Flash tester	1.057	1.145	1.092	1.692
Aperture Ø 2 (mm)	Flash tester	1.965	2.095	1.907	2.296
Aperture Ø 9.5 (mm) inlet of nozzle	Flash tester	9.666	10.063	9.795	11.065
Ø 1, Ø 2 coaxiality, measured by center-to-center distance method	Flash tester	0.072	0.115	0.074	0.252
Ø 9.5, Ø 2 coaxiality, measured by center-to-center distance method	Flash tester	0.113	0.125	0.120	0.303
Ø 1, Ø 2, Ø 9.5 coaxiality, measured by center-to-center distance method	Cumulative range estimation	0.185	0.240	0.194	0.555
According to the national standard GB/T 1184-1996	Ø 1, Ø 2 coaxiality level assembly 11 mm, IT12 ≤ 250 μm	IT9	IT11	IT9	IT12
	Ø 9.5, Ø 2 coaxiality level assembly 50 mm, IT12 ≤ 400 μm	IT9	IT12	IT11	IT12
	Ø 1, Ø 9.5, Ø 2 coaxiality level assembly 61 mm, IT12 ≤ 500 μm	IT10	IT11	IT11	IT12

**Table 6:** The percentage of metal contents in the wear area at the Nozzle A and Nozzle B exits.

Analysis Report of Figure 7c (Nozzle A)							Analysis Report of Figure 8c (Nozzle B)						
Elt.	Line	Intensity (c/s)	Atomic %	Atomic Ratio	Conc.	Units	Elt.	Line	Intensity (c/s)	Atomic %	Atomic Ratio	Conc.	Units
N	Ka	30.34	17.348	1.0000	5.115	wt.%	N	Ka	20.94	29.550	1.0000	9.922	wt.%
Al	Ka	7.26	0.647	0.0373	0.367	wt.%	Al	Ka	11.76	2.509	0.0849	1.623	wt.%
Si	Ka	20.92	1.416	0.0816	0.837	wt.%	Si	Ka	12.60	2.079	0.0703	1.400	wt.%
P	Ka	2.04	0.112	0.0064	0.073	wt.%	P	Ka	1.18	0.159	0.0054	0.118	wt.%
S	Ka	3.49	0.158	0.0091	0.107	wt.%	S	Ka	2.17	0.243	0.0082	0.186	wt.%
Ti	Ka	4.08	0.133	0.0077	0.134	wt.%	Ti	Ka	2.55	0.212	0.0072	0.243	wt.%
V	Ka	1.69	0.060	0.0035	0.064	wt.%	V	Ka	0.00	0.000	0.0000	0.000	wt.%
Cr	Ka	402.61	14.882	0.8579	16.289	wt.%	Cr	Ka	139.44	13.031	0.4410	16.242	wt.%
Mn	Ka	22.41	1.070	0.0617	1.238	wt.%	Mn	Ka	4.08	0.486	0.0165	0.640	wt.%
Fe	Ka	1030.27	58.536	3.3742	68.810	wt.%	Fe	Ka	327.69	46.265	1.5656	61.934	wt.%
Ni	Ka	69.05	5.638	0.3250	6.966	wt.%	Ni	Ka	27.18	5.467	0.1850	7.692	wt.%
Total			100.000		100.000	wt.%	Total			100.00		100.0	wt.%

**Table 7:** Statistical table of PV value and RMS value of BK7 before and after polished.

Nozzle Composite (wt.%)	Experiment Number	PV Value (nm)		RMS Value (nm)	
		Before Polished	After Polished	Before Polished	After Polished
Nozzle A: (304 stainless steel/2% titanium alloy)	1	320.695	210.558	80.596	30.685
	2	318.765	160.135	75.558	20.755
	3	310.258	170.568	70.586	15.734
Nozzle B: (304 stainless steel)	1	315.556	190.568	72.586	60.634
	2	330.560	200.356	76.556	58.544
	3	320.320	210.138	78.336	68.557

## Data Availability

The raw data supporting the conclusion of this article will be made available by the authors, without undue reservation.
